# Luteolin Prevents Cardiac Dysfunction and Improves the Chemotherapeutic Efficacy of Doxorubicin in Breast Cancer

**DOI:** 10.3389/fcvm.2021.750186

**Published:** 2021-10-13

**Authors:** Youyang Shi, Feifei Li, Man Shen, Chenpin Sun, Wei Hao, Chunyu Wu, Ying Xie, Shuai Zhang, Hongzhi Gao, Jianfeng Yang, Zhongyan Zhou, Dongwen Gao, Yuenong Qin, Xianghui Han, Sheng Liu

**Affiliations:** ^1^Longhua Hospital, Shanghai University of Traditional Chinese Medicine, Shanghai, China; ^2^School of Pharmacy, Shanghai University of Traditional Chinese Medicine, Shanghai, China; ^3^Department of Breast Surgery (Integrated Traditional and Western Medicine), Longhua Hospital, Shanghai University of Traditional Chinese Medicine, Shanghai, China; ^4^Department of Cardiovascular Research Laboratory, Longhua Hospital, Shanghai University of Traditional Chinese Medicine, Shanghai, China

**Keywords:** luteolin, cardiac dysfunction, doxorubicin, breast cancer, mitochondrial dysfunction

## Abstract

**Background:** Doxorubicin (Dox) is one of the most effective chemotherapy agents used in the treatment of solid tumors and hematological malignancies. However, it causes dose-related cardiotoxicity that may lead to heart failure in patients. Luteolin (Lut) is a common flavonoid that exists in many types of plants. It has been studied for treating various diseases such as hypertension, inflammatory disorders, and cancer. In this study, we evaluated the cardioprotective and anticancer effects of Lut on Dox-induced cardiomyopathy *in vitro* and *in vivo* to explore related mechanisms in alleviating dynamin-related protein (Drp1)-mediated mitochondrial apoptosis.

**Methods:** MTT and LDH assay were used to determine the viability and toxicity of cardiomyocytes treated with Dox and Lut. Flow cytometry was used to examine ROS levels, and electron and confocal microscopy was employed to assess the mitochondrial morphology. The level of apoptosis was examined by Hoechst 33258 staining. The protein levels of myocardial fission protein and apoptosis-related protein were examined using Western blot. Transcriptome analysis of the protective effect of Lut against Dox-induced cardiac toxicity in myocardial cells was performed using RNA sequencing technology. The protective effects of Lut against cardiotoxicity mediated by Dox in zebrafish were quantified. The effect of Lut increase the antitumor activity of Dox in breast cancer both *in vitro* and *in vivo* were further employed.

**Results:** Lut ameliorated Dox-induced toxicity in H9c2 and AC16 cells. The level of oxidative stress was downregulated by Lut after Dox treatment of myocardial cells. Lut effectively reduced the increased mitochondrial fission post Dox stimulation in cardiomyocytes. Apoptosis, fission protein Drp1, and Ser616 phosphorylation were also increased post Dox and reduced by Lut. In the zebrafish model, Lut significantly preserved the ventricular function of zebrafish after Dox treatment. Moreover, in the mouse model, Lut prevented Dox-induced cardiotoxicity and enhanced the cytotoxicity in triple-negative breast cancer by inhibiting proliferation and metastasis and inducing apoptosis.

## Introduction

Doxorubicin (Dox), an anthracycline chemotherapeutic agent, has been widely used to treat a variety of tumors including breast cancer, ovarian cancer, and hematological malignancies (1–4). However, the clinical utility of Dox in chemotherapy is limited by its adverse dose-dependent cardiotoxicity, which often results in left ventricular dysfunction, cardiomyopathy, and even heart failure (5, 6). Over the decades, novel insights into Dox-induced oxidative stress in cardiomyocytes emerged since current interventions to lessen the incidence of cardiotoxicity after prolonged Dox treatments are unsatisfactory (7–9). Increasing evidence proved that Dox facilitates cardiomyocyte apoptosis and programmed death by damaging mitochondrial structure and its biologic function, which is ascribed to the disorder of mitochondrial oxidation-reduction homeostasis and mitochondrial dynamics (10). Nevertheless, effective interventions for Dox-induced cardiotoxicity still need to be explored and developed.

Dexrazoxane is the only drug currently approved by the FDA that provides protection against Dox-induced cardiotoxicity. However, dexrazoxane not only causes side effects, such as hematological toxicity and myelosuppression, but also decreases the antitumor efficacy of Dox (11, 12). For instance, the activation of hypoxia-inducible transcription factor, an oncogene, may contribute to the protective effect of dexrazoxane against anthracycline cardiotoxicity in dexrazoxane-treated H9c2 cardiomyocytes (13). Interestingly, numerous studies have demonstrated that different herbal products and bioactive phytochemicals could counterbalance Dox-induced cardiotoxicity as add-on therapies (14, 15). Therefore, developing a drug that confers cardioprotection during Dox treatment and improves the chemotherapeutic efficacy of Dox in cancer cells is important.

**Figure 1:**
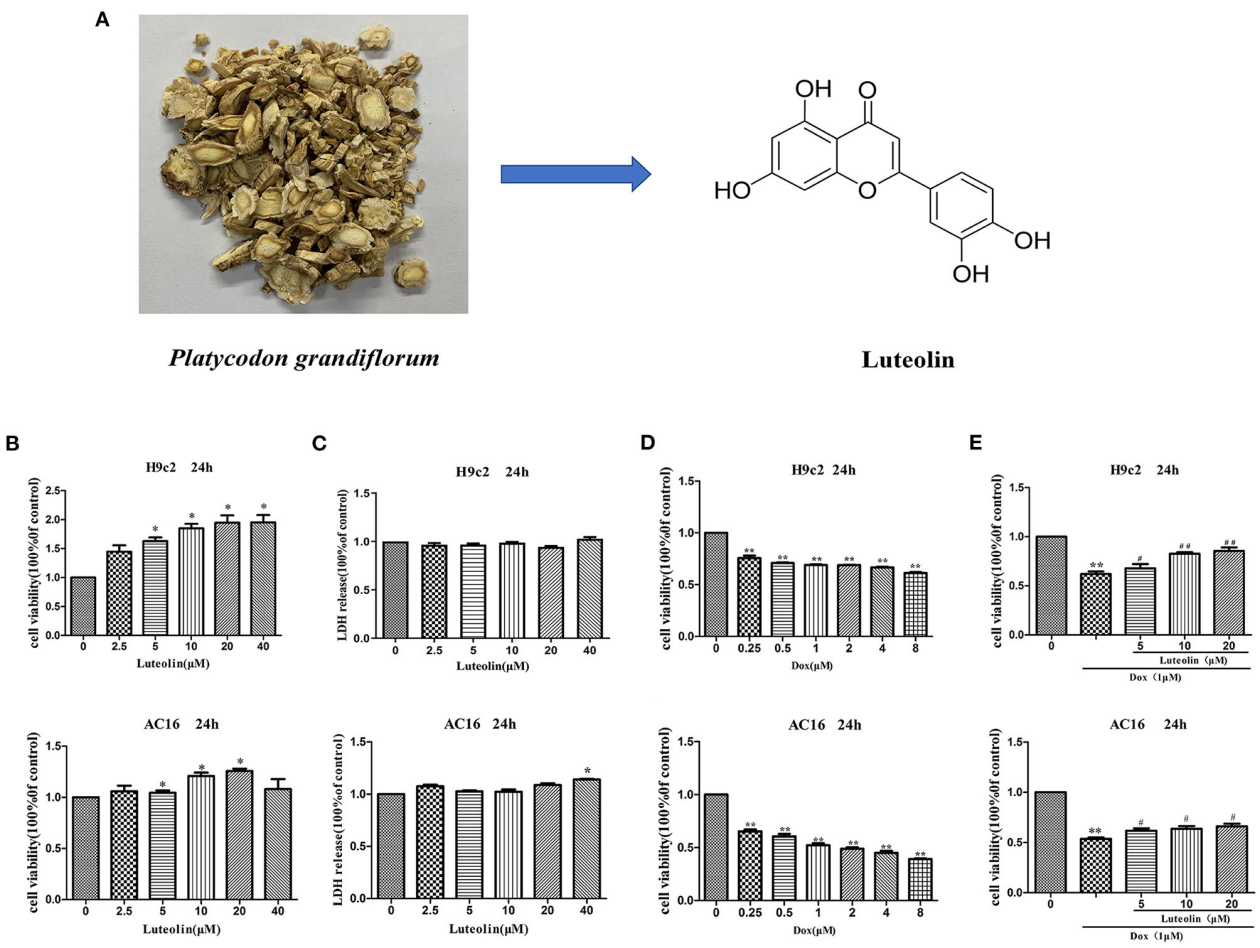
Effect of Lut in attenuating Dox-induced cardiotoxicity in H9c2 and AC16 cells. **(A)** Chemical structure of Lut. **(B,C)** Effects of different concentrations of Lut on cell viability and toxicity in H9c2 and AC16 cells. **(D)** Effects of different concentrations of Dox on cell toxicity in H9c2 and AC16 cells. **(E)** Effects of Lut in attenuating Dox-induced cardiotoxicity in H9c2 and AC16 cells. Mean ± SD, *n* = 3 independent experiment. ^*^P < 0.05; ^**^P < 0.01 compared with control group. ^#^*P* < 0.05; ^##^*P* < 0.01 compared with Dox group.

Luteolin (Lut), 3′,4′,5′,7′-tetrahydroxyflavone, a naturally occurring flavone, which are widely enriched in plants. Lut has shown beneficial effects in several biological processes including anti-tumorigenesis, anti-inflammation, antiapoptotic activities, and antioxidative stress (Figure 1A) (16, 17). Plants rich in Lut have been used as traditional Chinese medicine (TCM) for hypertension, inflammatory diseases, and cancers (14, 18). In China, traditional herbal medicine has been commonly used for the treatment of breast cancer and its complications (19). Among them, *Platycodon grandiflorum* is widely used, alone or in combination with other herbal medicines, to treat patients with early breast cancer receiving anthracycline-based chemotherapy. Our previous clinical study found that *Platycodon grandiflorum* has cardioprotective effects for early breast cancer patients who received Dox-based chemotherapy (20). Basic experiment studies revealed that *Platycodon grandiflorum* prevents Dox-induced cardiotoxicity in a mouse model of breast cancer (21). However, the potential mechanisms behind the cardioprotective effects remain unknown.

Lut is one of the major metabolites upon oral administration of luteolin-7-O-glucoside and is generally absorbed by intestinal mucosa into the systemic circulation after oral administration with an oral bioavailability at ~26% (22). Importantly, the flavonoid Lut is recognized as an important regulator of myocardial function providing myocardial protection during times of stress and can largely protect the myocardium against IR injury, partly through the downregulation of antioxidant and apoptosis properties (23, 24). Importantly, as the main component of *Platycodon grandiflorum*, Lut exerts multiple cellular effects *in vitro*, including antiproliferative effects in cancer cells and anti-inflammatory and antioxidative effects in various cell types. However, the molecular mechanisms by which Lut exerts these effects remain unclear.

Previous studies shown that Dox may activate apoptotic signaling through multiple mechanisms, including mitochondria-related apoptotic signaling (25). Dox-induced mitochondrial fission is a dynamin-related protein 1 (Drp1) signaling-dependent process, Drp1 might be a potential target against Dox-induced cardiotoxicity (26, 27). Given that hepatotoxicity and heart failure due to different medicines and toxins can be attenuated by Lut, we hypothesized that Lut may have protective effects on cardiotoxicity due to Dox via regulating mitochondrial damage. Therefore, the aim of this work was to investigate the protective effect of Lut against Dox-induced cardiotoxicity. The results showed that this protection was mediated through Drp1-regulated mitochondria-related apoptosis both *in vitro* and *in vivo*. In addition, Lut enhanced the chemotherapeutic efficacy of Dox in breast cancer.

## Materials and Methods

### Cell Cultures

H9c2 (rat cardiomyocytes), AC16 (human cardiomyocytes), 4T1 (mouse breast cancer cell), and MDA-MB-231 (human breast cancer cell) cell lines were purchased from the Cell Bank of the Chinese Academy of Sciences (Shanghai, China). H9c2, AC16, and MDA-MB-231 cells were maintained in DMEM medium supplemented with 10% (v/v) FBS, 100 U/mL penicillin, and 100 mg/L streptomycin. The 4T1 cells were maintained in RPMI 1640 medium supplemented with 10% (v/v) FBS, 100 U/mL penicillin, and 100 mg/L streptomycin. The cells were incubated at 37°C in a 5% CO_2_ incubator with saturated humidity.

### Cell Viability and Cytotoxicity Assays

The cell viability and cytotoxicity of H9c2 and AC16 cells were detected by MTT assay and LDH assays. Briefly, the cells were plated in 96-well plates at a density of 5,000 cells/well, incubated overnight, and then exposed to 1 μM Dox with or without various concentrations of Lut for another 24 h. Cells were supplemented with 20 μL MTT and incubated for 4 h at 37°C. The formazan crystals that formed were subsequently dissolved in 150 μL DMSO, and the OD490 values were measured with a BioTek instrument (Winooski, Vermont, USA). For cytotoxicity assay, the release of LDH into the medium was determined using a Cytotoxicity Detection Kit (Beyotime, Shanghai, China). The absorbance was measured with a microplate reader at 490 nm.

### Oxidative Stress Analysis

After 24 h of Dox (1 μM) treatment with or without Lut (20 μM), H9c2 and AC16 cells were loaded with 10 μM DCFH-DA in medium for 30 min at 37°C. After incubation, the ROS levels were measured using a flow cytometer. For SOD analysis, cell supernatants were collected by centrifugation after treatment. The solution was measured by the WST-8 method according to the manufacturer's instructions. The SOD activity was presented as percent inhibition of the reduction of the chromogenic substrate.

### Cell Microfilament Cytoskeleton Staining

H9c2 and AC16 cells were seeded into 6-well plates. After 24 h of Dox (1 μM) treatment with or without Lut (20 μM), cells were fixed with 4% paraformaldehyde in PBS for 15 min. Suitable media were washed twice with wash buffer and permeabilized with 0.1% Triton X-100 in PBS for 5 min at room temperature. Following two washes with wash buffer, cells in suitable media were covered with dilute FITC-conjugated phalloidin in PBS immediately prior to use and incubated for 30 min to stain the actin. Nuclei counterstaining was performed by incubating cells with 0.1 μg/mL DAPI for 15 min. Fluorescence images were captured with a laser scanning confocal microscope.

### Cell Apoptosis Analysis

H9c2 and AC16 cells were seeded in 6-well flat-bottom microtiter plates at an initial cell density of 10^5^ cells/well and cultured overnight. After 24 h of Dox (1 μM) treatment with or without Lut (20 μM), cells were incubated with fresh medium containing 0.1 mmol/L Hoechst 33258 (Beyotime, Shanghai, China) in the dark for 10 min. The cells were washed three times with PBS, and the apoptotic cells were observed under a fluorescence microscope (Olympus, Tokyo, Japan).

### Western Blot

Western blot was used to evaluate the apoptosis-related protein in cells. Primary rabbit antibodies, such as Bax (#2772, 1:1,000), Bcl-2 (#3498, 1:1,000), Bcl-XL (#2764, 1:1,000), Caspase-3 (#9662, 1:1,000), Cleaved Caspase-3 (#9664, 1:1,000), β-actin (#3700, 1:1,000), GAPDH (#5174, 1:1,000), Drp1 (#8570, 1:1,000), phospho-Drp1 (Ser616) (#3455, 1:1,000), and horseradish peroxidase (HRP)-conjugated secondary antibody (#7074s, 1:5,000) were purchased from Cell Signaling Technology, Inc. (Beverly, MA, USA). Cells were washed with PBS for three times and lysed with lysis buffer. After incubation on ice for 30 min, the lysates were centrifuged at 12,000 g for 15 min at 4°C. Protein sample was denatured at 100°C for 10 min, separated by sodium dodecyl sulfate–polyacrylamide gel electrophoresis, and then transferred to PVDF membrane (Millipore). The membrane was incubated with the primary antibodies overnight. Then, the membrane was washed and incubated with secondary HRP-conjugated goat anti-rabbit or anti-mouse antibodies. Finally, the blots were developed with Enhanced ECL System (Beyotime, Shanghai, China), and the signal was quantified by Quantity One software (Bio-Rad).

### Confocal Microscopy and Electron Assessment on Mitochondrial Morphology

Mitochondrial morphology was assessed by confocal microscopy. After 24 h of Dox (1 μM) treatment with or without Lut (20 μM), the media were removed from the dish, and staining solution containing MitoTracker probe (Yeasen Biotech, Shanghai, China) was added. The lyophilized MitoTracker was dissolved in anhydrous dimethyl sulfoxide to a final concentration of 100 nmol/L and incubated for 30 min. Images were captured with a laser scanning confocal microscope (Olympus, Tokyo, Japan). Following Dox treatment with or without Lut, the H9c2 and AC16 cells were washed with PBS, collected, and fixed in 2.5% glutaraldehyde for over 2 h at 4°C. The specimens were subsequently rinsed with PBS, fixed in 1% osmium tetroxide for 1–2 h, and then dehydrated sequentially in graded concentrations of 50, 70, 80, 90, and 100% ethanol for 15 min. The specimens were then processed for Epon™ embedding and observed under a transmission electron microscope (CM100, Philips, Netherlands).

### Molecular Docking

Molecular docking was used to interpret the binding area of small molecule ligands and macromolecular receptors through computer simulation and then calculate the physical and chemical parameters for predicting the affinity between the two. The mol2 format of the active ingredient was downloaded from the PubChem database. Its energy was minimized through Chem3D and converted into pdb format. Small molecule compounds were imported into AutoDock Tools-1.5.6 software. Water molecules were deleted, atomic charges were added, and atom type was allocated. All flexible keys can be rotated by default and finally saved as a pdbqt file. The PDB format file of the crystal structure of the target was downloaded from the PDB database (Protein Data Bank). Pymol 2.3 software was used to delete irrelevant small molecules in the protein molecule. Then, we imported the protein molecule into the AutoDock Tools-1.5.6 software to delete water molecules and add hydrogen atom, and finally saved it as a pdbqt file. The processed active ingredient is a small molecule ligand, and the protein target is used as a receptor. The center position and length, width, and height of the Grid Box were determined according to the interaction site of the small molecule and the target. Finally, batch docking was carried out through AutoDock vina and python script. In analyzing the molecular docking results, we visualized the binding effect of compounds and proteins using Pymol 2.3 software.

### RNA Sequencing

H9c2 and AC16 cells were harvested after drug treatment (three samples per group). The total RNA of each sample was extracted using TRIzol (Thermo Fisher). The quality of the RNA was measured by the Agilent 2100 Bioanalyzer with the RNA 6000 Nano Kit (Agilent, Santa Clara, CA, USA). The RNA concentration, RIN value and fragment length distribution were analyzed. Construction of the sequencing library and RNA sequencing were performed by Sangon (Shanghai, China) using the Illumina NovaSeq Platform.

### Identification of Differentially Expressed Genes (DEGs) and Functional Enrichment Analysis

Limma package (version 3.40.2) of R software was used to screen out the DEGs in the Dox–Lut group compared with Lut-treated group and Dox-treated group compared with control group in H9c2 and AC16 cells. “Adjusted *P* < 0.05 and Log (Fold Change| > 1)” were defined as the cutoff for the identification of differentially expressed mRNAs. To further confirm the underlying function of potential targets, the data were analyzed by functional enrichment. Gene Ontology (GO) is a widely used tool for annotating genes with functions, especially molecular function (MF), biological pathways (BP), and cellular components (CC). Kyoto Encyclopedia of Genes and Genomes (KEGG) enrichment analysis is a practical database for analytical study of functional annotations and associated high-level genome-wide pathways. The results of functional enrichment are displayed in bubble charts.

### Zebrafish Maintenance and Drug Treatment

Tg (cmlc2: GFP) zebrafish with GFP specifically expressed in myocardial cells were used in this study. Zebrafish were maintained as described in the Zebrafish Handbook (28). All animal experiments were approved by the Animal Research Ethics Committee of Shanghai University of Traditional Chinese Medicine. Pair-wise mating (6–12 months old) was used to generate the zebrafish embryos, which were maintained in embryo medium at 28.5°C. All embryos were then raised in the embryo medium containing 1-phenyl-2-thiourea (200 mM) after 48 hpf. Zebrafish (2 dpf) at the same developmental stage were distributed into a 24-well microplate (5 fish per well). After co-treatment with Dox (10 μM) and different concentrations of Lut (5, 10, 20 μM) for 24 h, ventricular functions of zebrafish were examined by assessing various parameters and morphology. The morphology and functions of zebrafish heart were measured by an imaging system comprising a microscope (Olympus). Zebrafish were placed into 1% low-melting-point agarose (Gibco) to restrict their movement, and videos of zebrafish heartbeat were recorded for 10 s at room temperature. The parameters and morphology of ventricular function of zebrafish were measured.

### Wound Healing Assay

Cells were seeded in 6-well plates at a density of 1 × 10^5^ cells per well, and when cellular confluence reached about 90%, a 200 μL pipette tip was used to create wounds in confluent cells. After removing the floating cells by washing the scraped surface with PBS, wounded monolayers were photographed with a microscope. Cells were then incubated containing Dox (2 μM) with or without Lut (40 μM) for 24 h. The images of cells migrating into the wound surface and the average distance of migrating cells were determined under a microscope 24 h later.

### Colony Formation Assay

To further determine the inhibitory effect of Lut on the tumorigenicity of triple-negative breast cancer (TNBC) cells, colony formation assays were performed. Five hundred 4T1 or MDA-MB-231 cells were seeded into 6-well plates to incubate overnight. The cells were then incubated with Dox (2 μM) with or without Lut (40 μM) for 7–10 days. After fixing with 4% paraformaldehyde and staining with a crystal violet solution, colonies containing more than 30 individual cells were counted under a stereomicroscope.

### Cell Invasion Assay

The invasive ability of 4T1 and MDA-MB-231 cells were measured using 24-well Transwell with polycarbonate filters (pore size, 8 μm) coated on the upper side with Matrigel (BD, Bedford, MA, USA). 1 × 10^3^ cells in 100 mL medium were seeded in the top chamber. The bottom chamber contained 10% fetal calf serum medium. After 24 h incubation, non-invasive cells were removed with a cotton swab. Cells that migrated to the bottom surface of the membrane were fixed in formaldehyde, stained with crystal violet solution, and counted under a microscope.

### Xenograft Mouse Experiments

Seven-week-old female BALB/c mice (18–20 g) were obtained from the Shanghai SLAC Laboratory Animal Technology Co., Ltd. (Shanghai, China). The animals were housed under standardized conditions in animal facilities at 20 ± 2°C temperature, 40% ± 5% relative humidity, and a 12-h light/dark cycle with dawn/dusk effect. The protocol was approved by the Animal Research Ethics Committee of Shanghai University of Traditional Chinese Medicine (Permit Number: PZSHUTCM18122103). 4T1 cells (2 × 10^6^) were resuspended in 10 mL PBS, and 100 μL of cell suspension was subcutaneously injected into the second pair of breast fat pads on the left side of each mouse. The tumors formed approximately 14 days after the inoculation. Then, all mice were randomly divided into three groups (*n* = 5): control group (ip, saline), Dox group (ip, 2.5 mg/kg Dox), and Dox combined with Lut group (ip 2.5 mg/kg Dox + ip 30 mg/kg Lut). The mice were administered with Dox or Dox combined with Lut solution once per 2 days continuously for 2 weeks. At the experimental endpoint, all animals were euthanized. Then, the size and weight of tumors were measured. Lungs and tumors were excised and then fixed in 4% paraformaldehyde overnight until further analysis. For echocardiographic studies, the mice were anesthetized with 2.5% isoflurane in 95% oxygen and 5% carbon dioxide and then situated in the supine position on a warming platform to maintain the core temperature at 37°C. Cardiac function was evaluated via echocardiography by using a High-Resolution Small Animal Imaging System (Vevo2100, Visual Sonics Inc., Toronto, Canada). Two-dimensional and M-mode echocardiographic images of the long and short axis were recorded. Left ventricular ejection fraction (LVEF) and left ventricular fractional shortening (LVFS) were measured and calculated using the Vevo Strain Software Work Station.

### Statistical Analysis

All results are presented as mean ± standard deviation (SD). Two-tailed analysis of variance followed by Dunnett's *post hoc* test and Fisher's test was used to determine the statistical significance. *P* < 0.05 was considered significant for all tests.

## Results

### Lut Attenuates Dox-Induced Cardiotoxicity in H9c2 and AC16 Cells

The H9c2 (rat) and AC16 (human) cardiomyocytes were treated with elevated concentration (0, 2.5, 5, 10, 20, and 40 μM) of Lut for 24 h. As shown in Figure 1B, cell viability was markedly increased with Lut (*P* < 0.05). As detected by LDH assay, the increased Lut concentration was not significantly correlated with LDH release until the Lut concentration was increased to 40 μM (*P* < 0.05; Figure 1C). Dox (0, 0.25, 0.5, 1, 2, 4, and 8 μM) treatment for 24 h markedly decreased cell viability (*P* < 0.01; Figure 1D). Co-treatment with Lut and Dox significantly increased cell viability compared with Dox alone (*P* < 0.05; Figure 1E). Lut could significantly attenuate Dox-induced cardiotoxicity in H9c2 and AC16 cells.

### Lut Attenuates Dox-Induced Oxidative Stress and Cytoskeletal Damages in H9c2 and AC16 Cells

**Figure 2:**
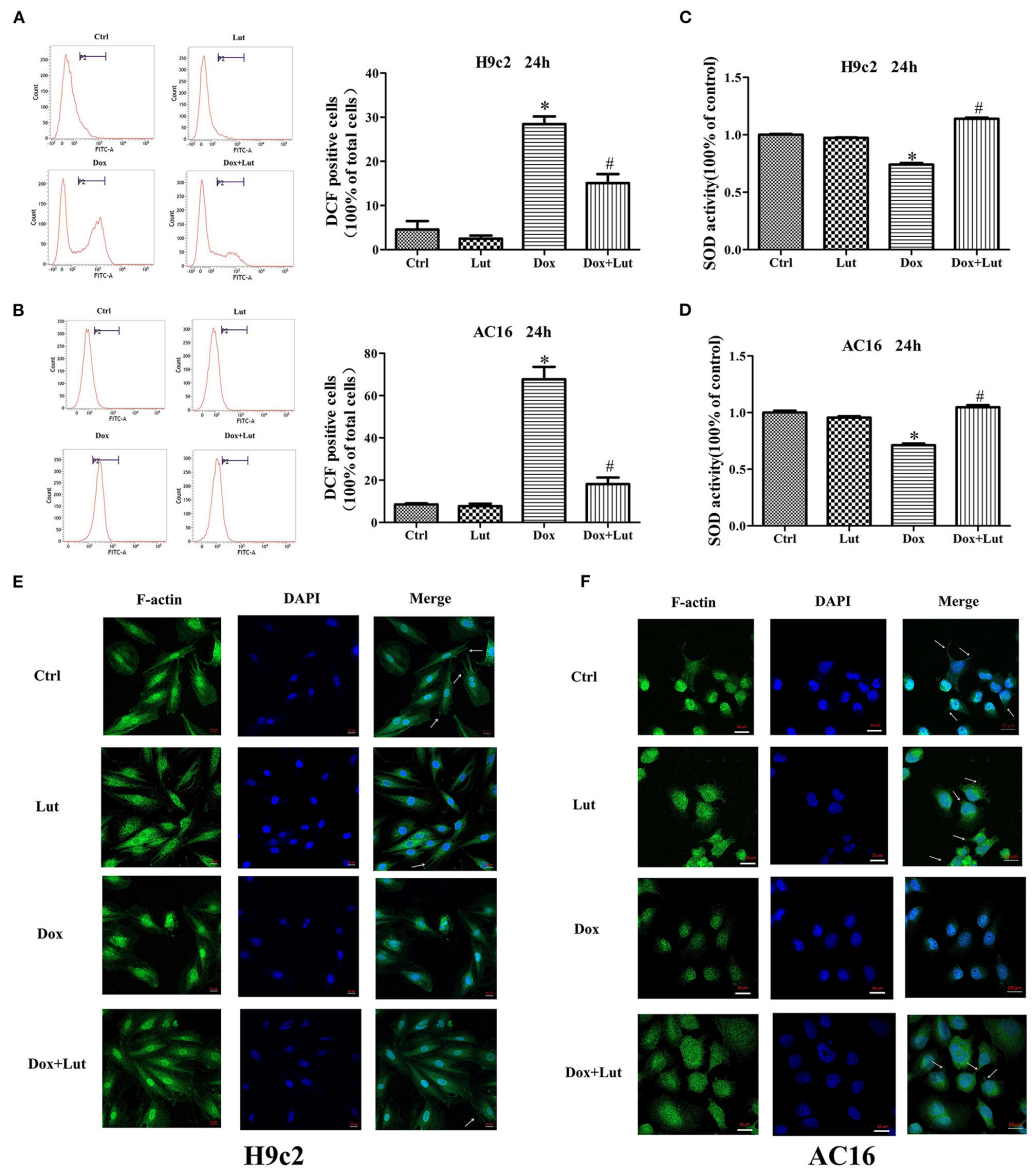
Effect of Lut treatment on Dox-induced oxidative stress and cytoskeletal damages in H9c2 and AC16 cells. **(A,B)** ROS and **(C,D)** SOD levels in H9c2 and AC16 cells after Dox and Lut treatment. **(E,F)** Cytoskeleton staining in H9c2 and AC16 cells after Dox and Lut treatment (630×). White arrows show microfilaments and microtubules. Mean ± SD, *n* = 3 independent experiment. ^*^P < 0.05 compared with control group. ^#^*P* < 0.05 compared with Dox group.

We then detected changes of oxidative stress in H9c2 and AC16 cells after 24 h drug treatments using flow cytometry. The results showed that Lut treatment did not significantly change the ROS level, but it could significantly reduce the elevated ROS level induced by Dox (*P* < 0.05; Figures 2A,B). Similarly, the decreased SOD activity induced by Dox could be significantly increased by Lut treatment, which could even reach a level higher than that in the Lut-alone group (*P* < 0.05; Figures 2C,D). The integrity of the myocardial cytoskeleton plays an important role in the physiological function of the heart. Interestingly, cytoskeleton staining suggested that the cytoskeleton of the Dox treatment group was damaged with disappearance of microfilaments and microtubules in the cell membrane and loss of cell fiber tension. However, this damage could be markedly recovered by Lut in the combined treatment group (white arrows, Figures 2E,F). In conclusion, Lut could significantly attenuate Dox-induced oxidative stress and restore cytoskeletal alterations in H9c2 and AC16 cells.

### Lut Inhibits Dox-Induced Cardiomyocyte Apoptosis in H9c2 and AC16 Cells

**Figure 3:**
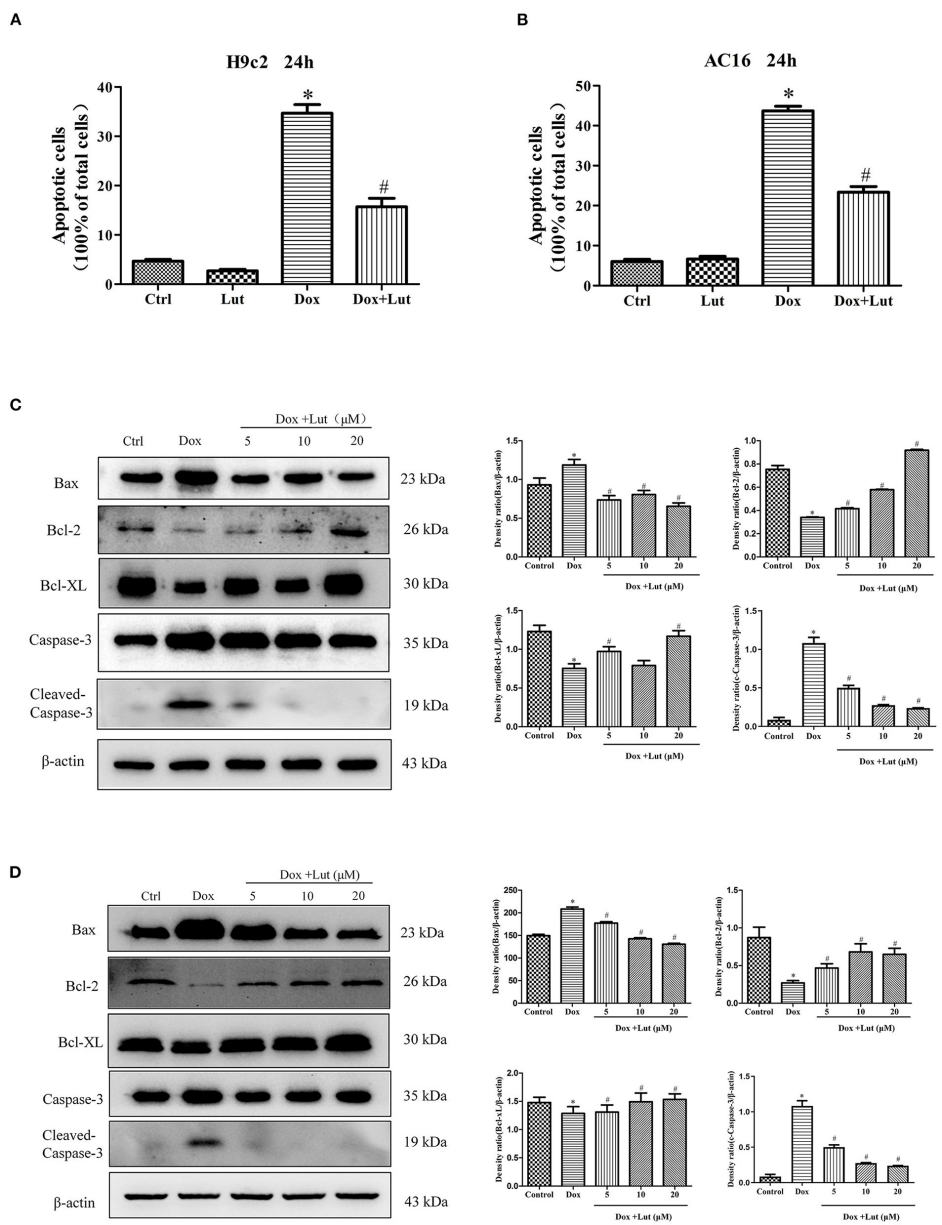
Effect of Lut treatment on Dox-induced cardiomyocyte apoptosis. **(A)** Quantified TUNEL-positive cells from three fields per group in H9c2 and **(B)** AC16 cells. **(C)** Representative Western blot images of H9c2 and **(D)** AC16 apoptosis using Bax, Bcl-2, Bcl-XL, and Cleaved Caspase-3. Mean ± SD, *n* = 3 independent experiment. ^*^P < 0.05 compared with control group. ^#^*P* < 0.05 compared with Dox group.

TUNEL assay was performed to assess apoptosis following Lut and Dox treatment in H9c2 and AC16 cells (Supplementary Figures 1A,B). Compared with the control group, Dox challenge for 24 h significantly increased cell apoptosis as evidenced by the elevated number of TUNEL-positive cardiomyocytes (*P* < 0.05), while the effect was significantly inhibited by Lut treatment (*P* < 0.05; Figures 3A,B). Meanwhile, Western blot indicated that Dox treatment upregulated the levels of Bax and Cleaved Caspase-3 and downregulated Bcl-2 and Bcl-XL levels in H9c2 and AC16 cells. Importantly, the regulation induced by Dox was conversely regulated by Lut treatment (*P* < 0.05; Figures 3C,D). Taken together, Lut treatment could significantly inhibit Dox-induced cardiomyocyte apoptosis through the Bax/Bcl-2/Caspase-3 pathway in H9c2 and AC16 cells.

### Lut Attenuates Dox-Induced Excessive Mitochondrial Division of H9c2 and AC16 Cells

**Figure 4:**
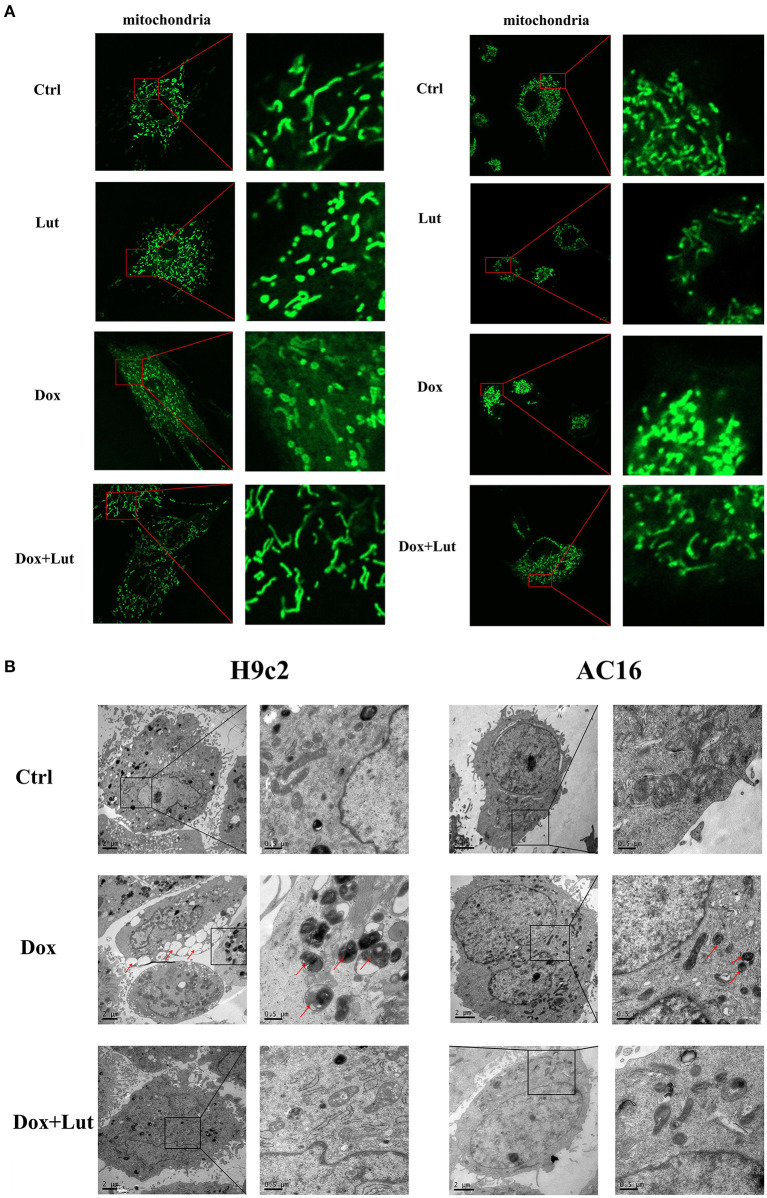
Effect of Lut treatment on Dox-induced changes in cardiomyocyte mitochondrial morphology. **(A)** Representative fluorescence images of the morphology of mitochondria in H9c2 (left) and AC16 (right) cells (630×). **(B)** Transmission electron microscopy images of the morphology of mitochondria in H9c2 (left) and AC16 (right) cells (12,000×). Red arrows show autophagosome.

Next, we explored the effect of Lut on the mitochondrial morphological change of cardiomyocytes induced by Dox. As shown in Figure 4A, fluorescence microscopy showed that the mitochondria of normal cardiomyocytes were reticulated. After being stimulated with Dox (1 μM) for 24 h, compared with the normal group, cell mitochondria were divided, and the morphology of cell mitochondria changed significantly, transforming from a reticulate to a punctate phenotype. In addition, compared with the Dox-treated group, Lut (20 μM) markedly inhibited the excessive division of mitochondria and restored the mitochondrial morphology of H9c2 and AC16 cells. Using transmission electron microscopy, we observed the ultrastructure of cells. After 24 h of Dox treatment, vacuoles appeared in cardiomyocytes, and a “hair ball” structure appeared in the mitochondria (red arrow, Figure 4B). After Lut treatment, the morphology of cell mitochondria was restored, and the morphology of cell nucleus and chromatin returned to normal.

### Lut Attenuates Dox-Induced Drp-1 Phosphorylation in H9c2 and AC16 Cells

**Figure 5:**
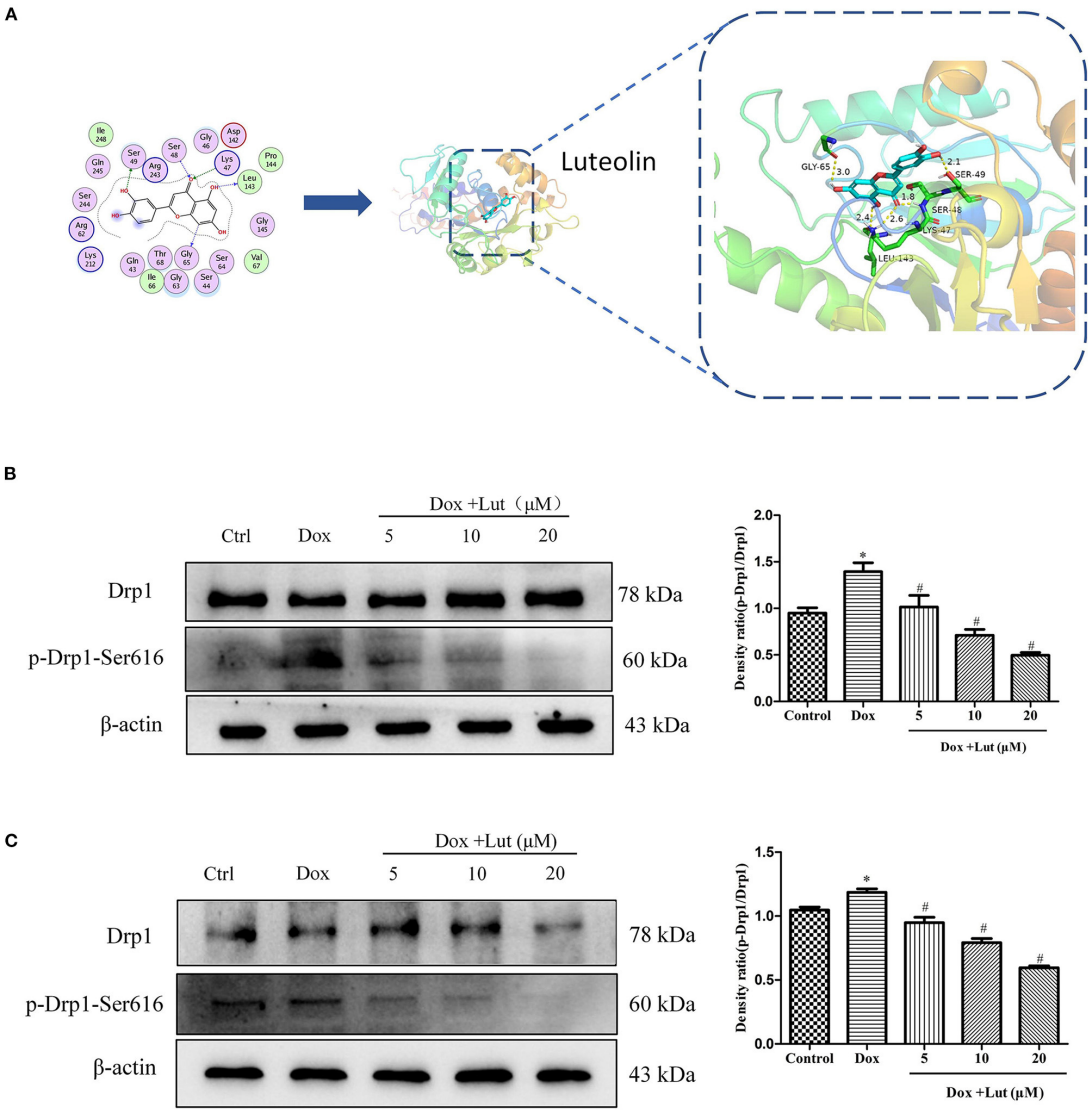
Lut attenuated Dox-induced Drp-1 phosphorylation in H9c2 and AC16 cells. **(A)** Detailed molecular docking simulations. The blue circle on the left represents the binding site of the small-molecule compound, and the bar graph on the right describes the specific form of this interaction. Representative Western blot images of Drp-1 and p-Drp-1 in H9c2 **(B)** and AC16 **(C)** cells. Mean ± SD, *n* = 3 independent experiment. ^*^P < 0.05 compared with control group. ^#^*P* < 0.05 compared with Dox group.

We tried to explore the mechanism of Lut to restore Dox-induced mitochondrial morphological alterations and used a molecular docking algorithm to predict the binding mode and affinity between the receptor and the drug molecule in Figure 5A. The results suggested a high affinity for docking between Drp-1 and Lut (affinity = −8.31 kcal/mol). Western blot revealed a significantly elevated p-Drp-1/Drp-1 ratio in the Dox-treated group, while the phosphorylation level of Drp-1 significantly decreased with additional Lut treatment in a dose-dependent manner compared with the Dox-treated group (*P* < 0.05; Figures 5B,C). Overall, Lut could significantly attenuate Dox-induced mitochondrial morphological changes *via* regulating Drp-1 phosphorylation in H9c2 and AC16 cells.

### Lut Reduces Heart Damage Induced by Dox *in vivo*

**Figure 6:**
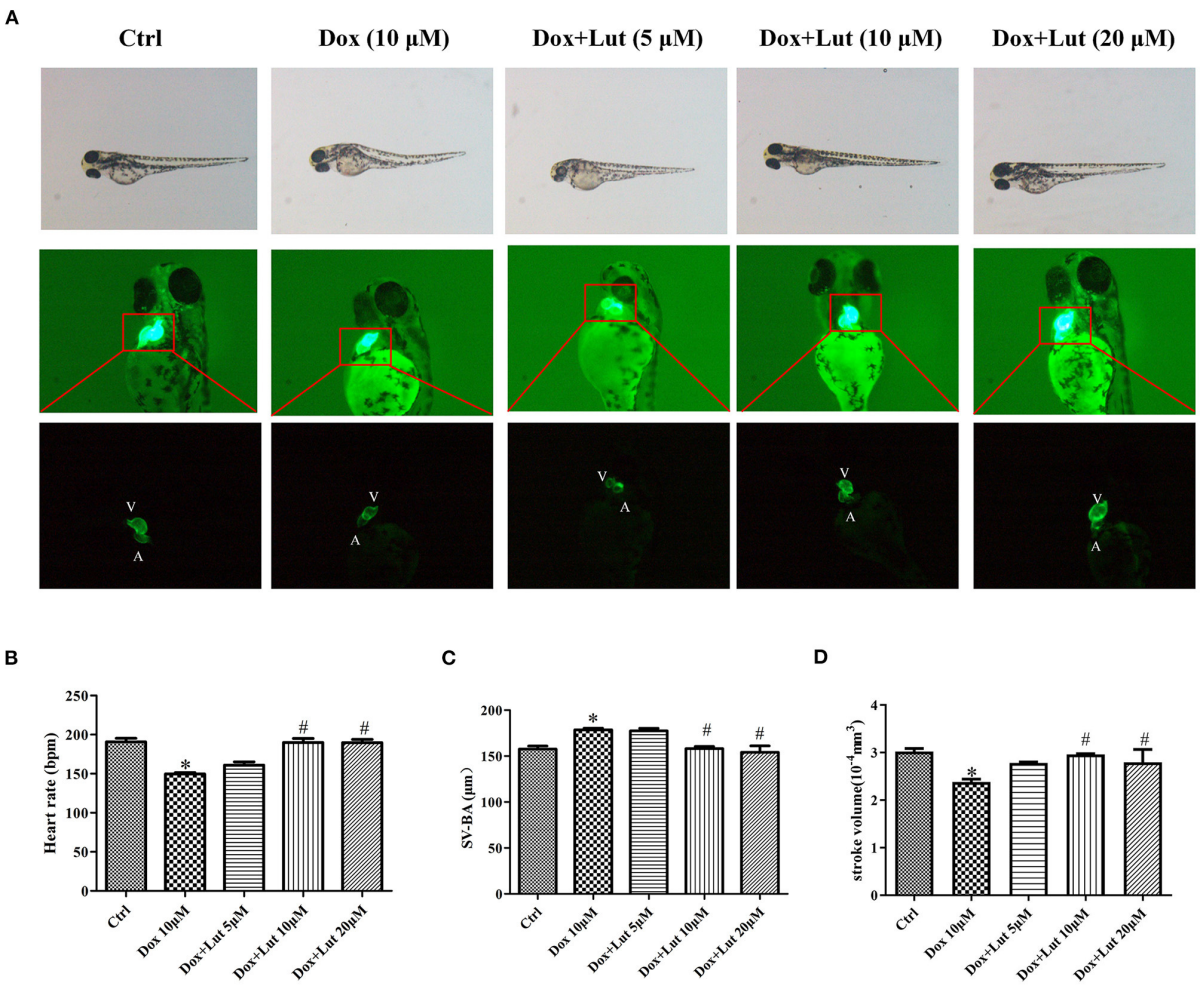
Lut protected the loss of ventricular function in zebrafish. **(A)** Representative images of zebrafish heart after treatment with Dox in the presence or absence of Lut. A: atrium, V: ventricle. Zebrafish were co-treated with Dox and Lut. The changes in **(B)** heart rate and **(C)** SA–BA **(D)** stroke volume were measured. Mean ± SD, *n* = 3 independent experiment. ^*^P < 0.05 compared with control group. ^#^*P* < 0.05 compared with Dox group.

The protective effects of Lut against cardiotoxicity mediated by Dox in zebrafish were quantified. As shown in Figure 6A, we constructed a zebrafish heart injury model using 10 μM Dox. After co-administration of Dox and different concentrations of Lut for 24 h, the zebrafish pericardium of the model group showed obvious edema compared with the negative control group. Moreover, we found a significantly decreased zebrafish heart rate, increased SV–BA distance, and decreased stroke volume in the Dox-induced group (*P* < 0.05; Figures 6B–D), indicating severe heart damage. Compared with the doxorubicin-induction group, we found significantly increased heart rate, shortened SV–BA distance, and markedly improved stroke volume of zebrafish after 24 h of intervention with medium and high doses of Lut (*P* < 0.05; Figures 6B–D).

### Lut Interferes With Dox-Induced Transcriptome Sequencing of Cardiomyocytes in AC16 and H9c2 Cells

**Figure 7:**
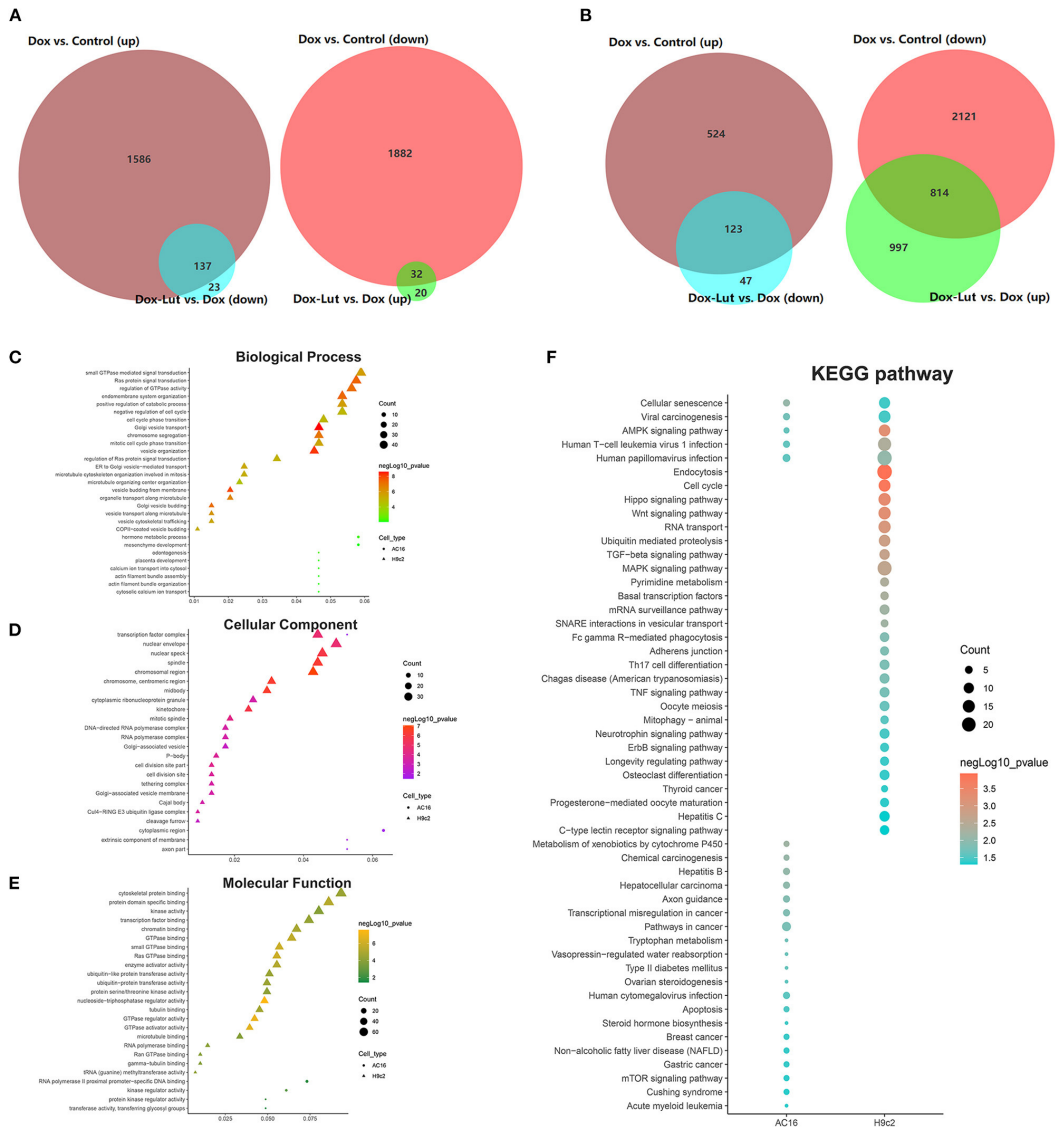
GO and KEGG analysis of DEGs in the Dox–Lut group compared with Dox group. **(A)** Venn diagram. The intersection in the figure is the gene with the opposite differential expression, which is defined as the gene affected by Lut in AC16 and **(B)** H9c2 cells. **(C)** Biological processes, **(D)** cellular component, **(E)** molecular function, and **(F)** KEGG pathways involved in resveratrol-affected genes.

Subsequently, to identify DEGs and hallmarks related to the process of Lut in attenuating the toxicity of Dox to cardiomyocytes, we used RNA sequencing and selected upregulated DEGs in the Dox group compared with control group and downregulated DEGs in the Dox–Lut group compared with Dox group. We screened out a total of 137 overlapped hub genes in AC16 cells and 123 overlapped hub genes in H9c2 cells (Figures 7A,B). Similarly, we identified downregulated DEGs in the Dox group compared with the control group and upregulated DEGs in the Dox–Lut group compared with the Dox group. Then, we screened out a total of 32 overlapped hub genes in AC16 cells and 814 overlapped hub genes in H9c2 cells (Figures 7A,B). Next, we explored the functional annotations of different genes in cardiomyocytes using GO and KEGG algorithm. The DEGs were significantly involved in biological process (GO: BP), including actin filament bundle organization, Golgi vesicle transport, Ras protein signal transduction, organelle transport along microtubule, microtubule organizing center organization, and microtubule cytoskeleton organization involved in mitosis; cellular function (GO: CC), including chromosomal region, mitotic spindle, P-body, Golgi-associated vesicle membrane, and cleavage furrow; and molecular function (GO: MF), including kinase regulator activity, GTPase activator activity, tubulin binding, cytoskeletal protein binding, and microtubule binding (Figures 7C–E). Additionally, DEGs of AC16 and H9c2 cells significantly participated in cellular senescence, AMPK signaling pathway, viral carcinogenesis, and human T-cell leukemia virus 1 papillomavirus infection, suggesting that drug-induced cellular senescence may increase the virus susceptibility and carcinogenicity of cardiomyocytes (Figure 7F). We found that the DEGs not only markedly participated in Hippo/Wnt, AMPK/MAPK, and TGF-β signaling pathways and animal mitophagy process, but were also involved in transcriptional misregulation and pathways in cancers, such as hepatocellular, breast, gastric, and thyroid cancer.

### Lut Promotes the Antitumor Effect of Dox in 4T1 and MDA-MB-231 Cells

**Figure 8:**
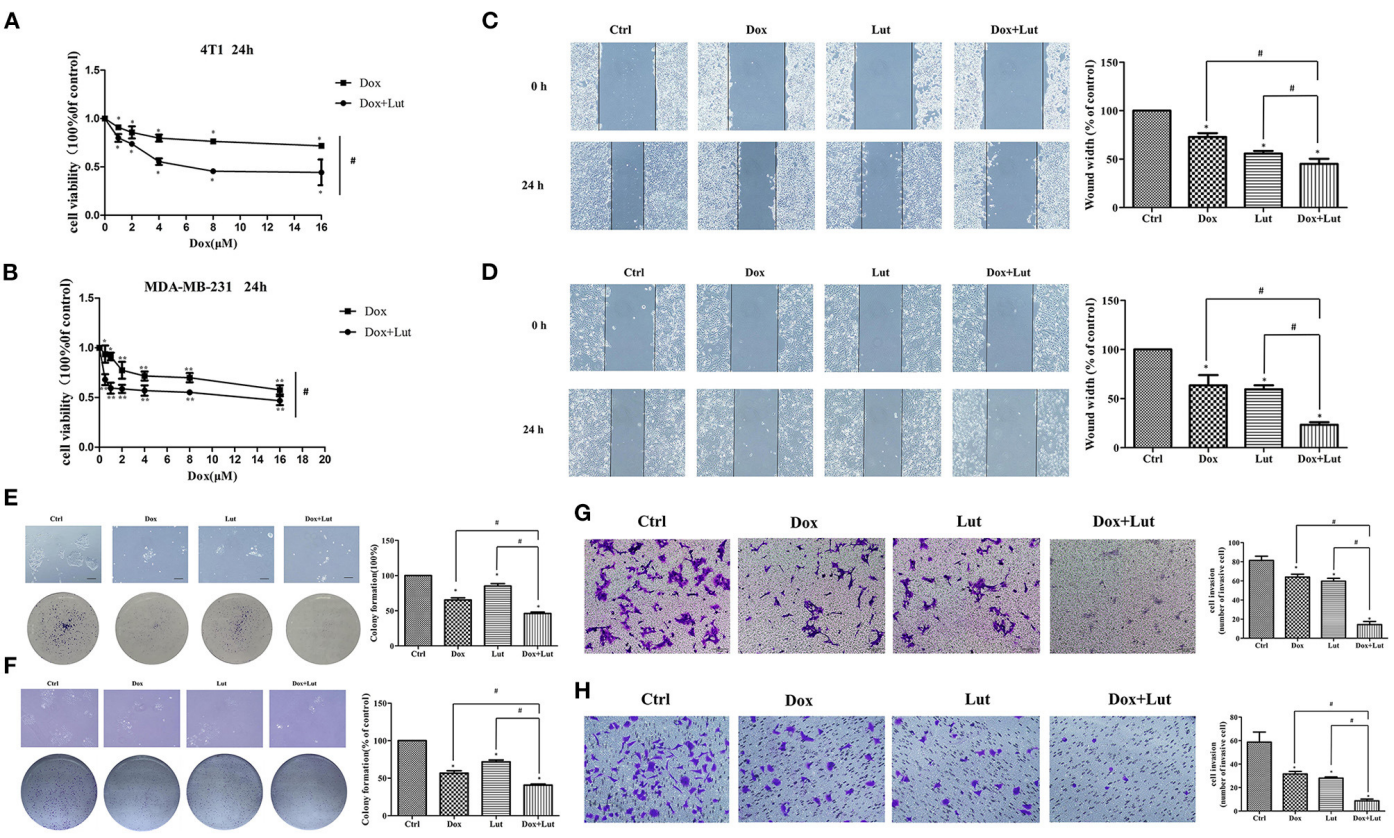
Lut promotes the antitumor effect of Dox in 4T1 and MDA-MB-231 cells. **(A)** 4T1 and **(B)** MDA-MB-231 cells were treated with Dox or Dox added with Lut (40 μM) at concentrations of 0, 1, 2, 4, 8, or 16 μM for 24 h. MTT assays were performed, and cell viability was determined. Photographs and quantification of wounds, colony formation, and cell migration to **(C,E,G)** 4T1 and **(D,F,H)** MDA-MB-231 cells treated with Dox (2 μM) or Dox added with Lut (40 μM). Mean ± SD, *n* = 3 independent experiment. ^*^P < 0.05 compared with control group. ^#^*P* < 0.05 compared with Dox group.

To further explore the effect of Lut on the antitumor efficacy of Dox, we explored the malignant biological behavior of different treatments in invasive TNBC 4T1 and MDA-MB-231 cell lines. As shown in Figures 8A,B, the cell viability was markedly decreased in the Lut-added group compared with the Dox-induced group in 4T1 and MDA-MB-231 cells (*P* < 0.05). Wound healing test showed significantly reduced wound width after 24 h of induction of Lut or Dox compared with the negative control group, while the combination of Lut and Dox remarkably decreased wound healing width compared with the single-drug treatment group (*P* < 0.05; Figures 8C,D). In addition, Lut significantly enhanced the antitumor efficacy of Dox by decreasing the colony formation and invasion ability of breast cancer cells (*P* < 0.05; Figures 8E–H). In general, Lut could not only significantly inhibit the malignant behavior of tumor cells, but also enhance the antitumor efficacy of Dox in 4T1 and MDA-MB-231 cells.

### Lut Promotes Dox-Induced Cell Apoptosis via the Bax/Bcl-2/Caspase-3 Pathway in 4T1 and MDA-MB-231 Cells

**Figure 9:**
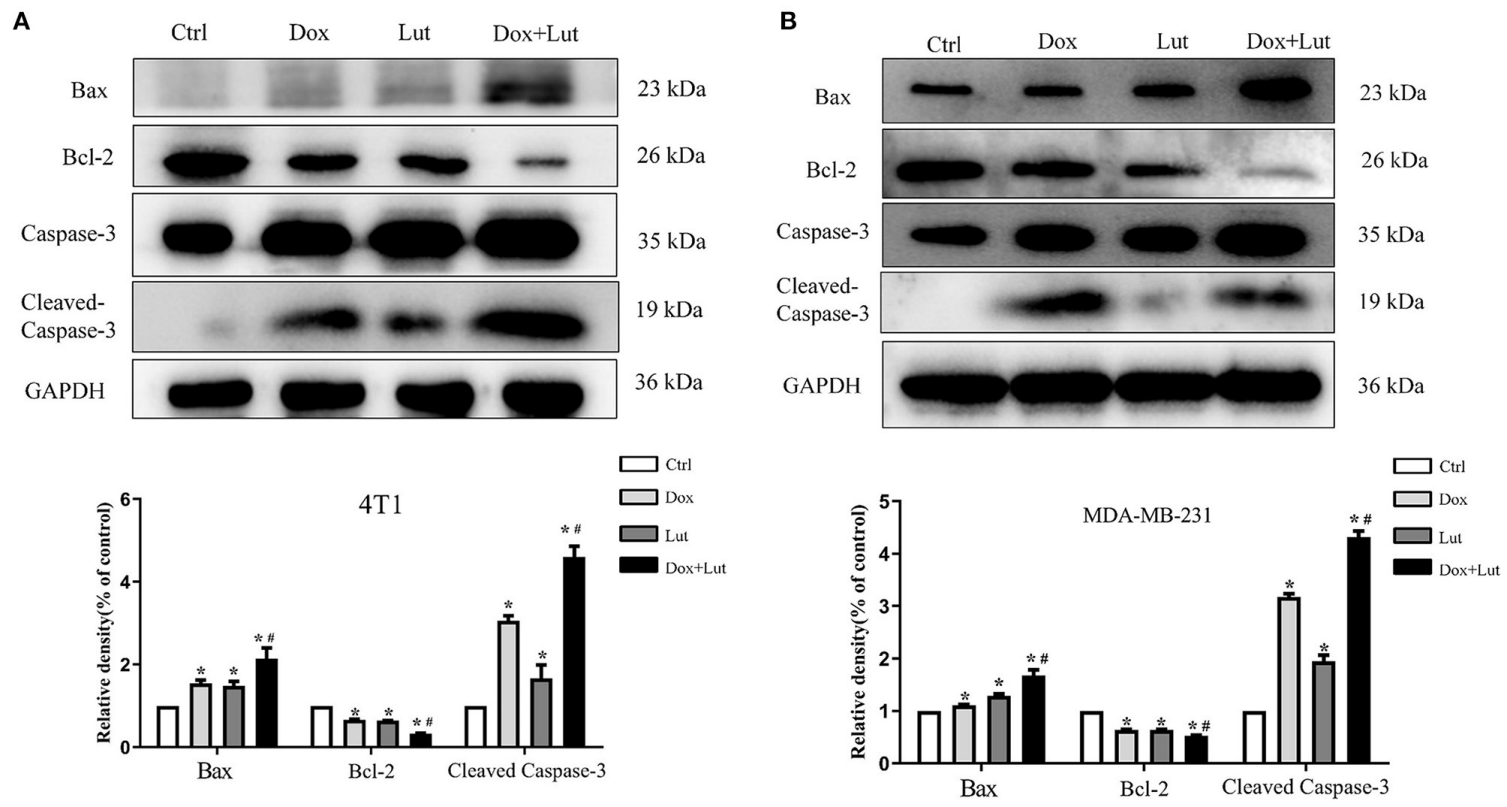
Lut promotes Dox-induced cell apoptosis *via* the Bax/Bcl-2/Caspase-3 pathway in TNBC cancer. Western blot images of Bax, Bcl-2, and Cleaved Caspase-3 expression in 4T1 **(A)** and MDA-MB-231 **(B)** cells after treatment with Dox (2 μM) or Dox added with Lut (40 μM) at the indicated concentrations for 24 h. Quantification of protein expression is shown below the Western blots. ^*^P < 0.05 compared with control group. ^#^*P* < 0.05 compared with Dox group.

Next, we explored the effect of Lut on the apoptosis of triple-negative breast cancer cells induced by Dox. Western blot indicated upregulated levels of Bax and Cleaved Caspase-3 in conjunction with downregulated Bcl-2 levels in Dox-treated or Lut-treated 4T1 and MDA-MB-231 cells. Importantly, the regulation of cell apoptosis induced by Dox was significantly enhanced by additional Lut treatment (*P* < 0.05; Figures 9A,B). Taken together, Lut treatment could significantly enhance Dox-induced tumor cell apoptosis through the Bax/Bcl-2/Caspase-3 pathway in 4T1 and MDA-MB-231 cells.

### Lut Prevents the Cardiotoxicity and Promotes the Antitumor Effect Induced by Dox *in vivo*

**Figure 10:**
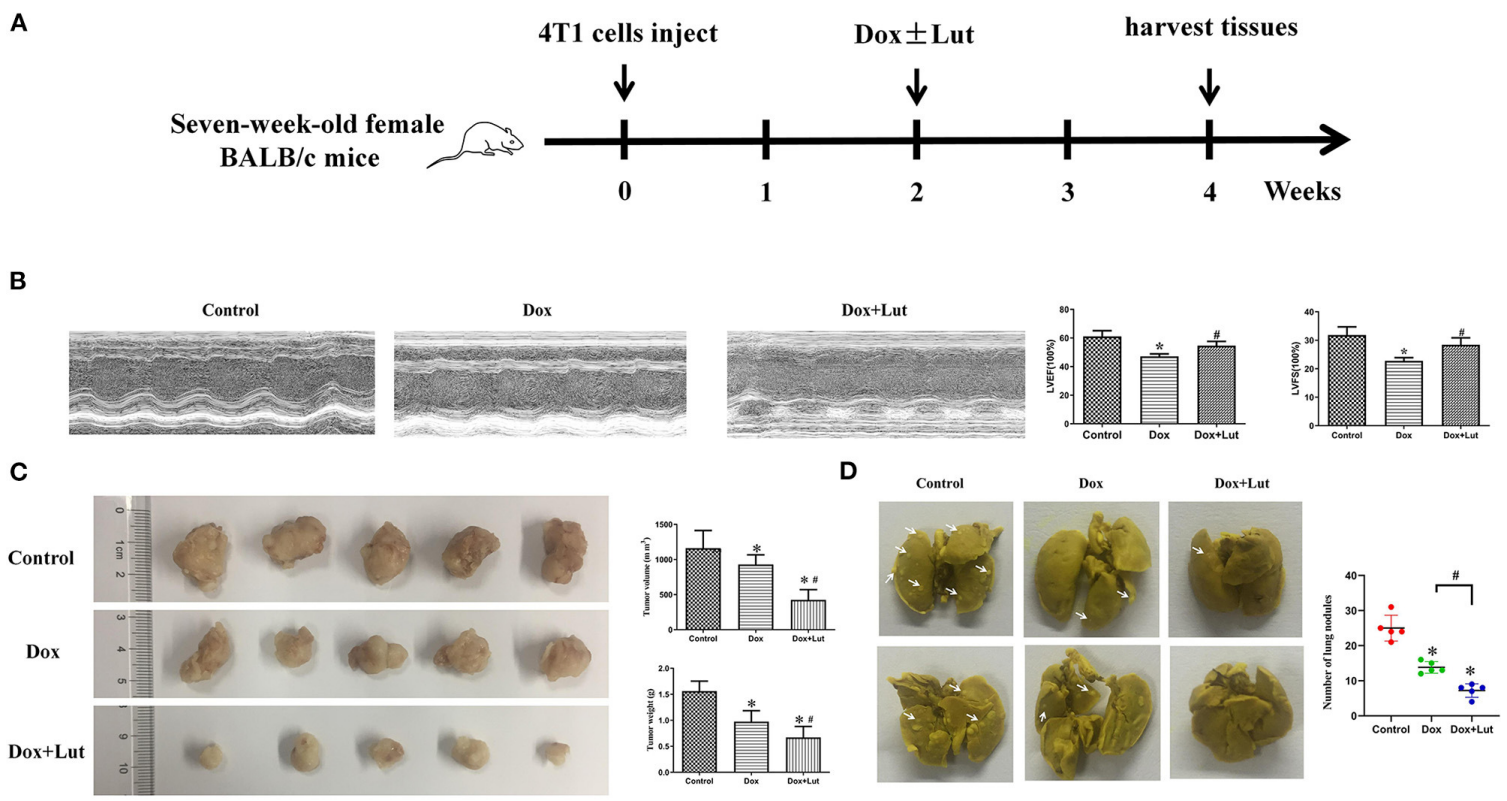
Lut prevents the cardiotoxicity and promotes the antitumor effect induced by Dox *in vivo*. **(A)** Diagram showing the scheme for tumor implantation and Lut treatment. **(B)** Echocardiographic assay was used to determine the attenuated left ventricular dysfunction of Lut on Dox-induced cardiac dysfunction in mice. **(C)** Image of tumors from different groups. The weight and size of the tumor was measured. **(D)** Typical lung nodules in mice from different groups. ^*^P < 0.05 compared with control group. ^#^*P* < 0.05 compared with Dox group.

A xenograft of 4T1 cells in 7-week-old BALB/c mice was established for *in vivo* exploration (Figure 10A). Echocardiographic examination showed that the Dox-treated group had an ~20% decrease in LVEF and LVFS compared with the control group. Lut treatment significantly attenuated cardiac dysfunction in the Dox-treated mice, as indicated by the increased LVEF and LVFS (*P* < 0.05; Figure 10B). Additionally, Dox did not alter the cardiac structure, including the diastolic left ventricular internal dimension (LVIDd), diastolic left ventricular posterior wall (LVPWd), and diastolic interventricular septum (IVSd) (Supplementary Figure 2). As shown in Figure 10C, the tumor volume and weight were significantly decreased in the Dox-induced group compared with the control group and was even further reduced in the Dox–Lut group (*P* < 0.05; Figure 10C). Notably, Lut also significantly enhanced the Dox-induced reduction of the number of lung metastatic nodules in xenograft models (*P* < 0.05; Figure 10D). Taken together, Lut could significantly promote the antitumor efficiency induced by Dox in a xenograft of highly aggressive 4T1 cells.

## Discussion

Breast cancer is one of the most prevalent malignancies and associated with significant morbidity among females worldwide (29). Among the treatments of primary breast cancer, an anthracycline-based regimen is the standard of care (29, 30). According to the latest National Comprehensive Cancer Network guidelines, 5-fluorouracil, epirubicin, and cyclophosphamide adjuvant chemotherapy regimen followed by paclitaxel or paclitaxel combined with anti-human epidermal growth factor-2 trastuzumab is the recommended regimen for breast cancer (31). Anthracyclines represented by Dox are the first-line chemotherapy for breast cancer, and they play an irreplaceable role in current clinical treatment of breast cancer. Unfortunately, the adverse effects of Dox, such as immunosuppression, hepatotoxicity, and especially dose-dependent cardiotoxicity, limit its efficacy and application because treatment-related cardiotoxic adverse events have become one of the common causes of breast cancer mortality (32, 33). Current prevention and treatment cannot effectively solve the problem of Dox-induced cardiotoxicity (34, 35). Therefore, improved approaches to reduce Dox side effects and enhance Dox efficiency need to be developed.

TCM becoming increasingly important in cancer treatment and modern cardiotoxicity protective pharmacology. The identification of cardiotoxic protective drugs with unique pharmacological effects from TCM has become a new direction (36). For example, Zheng et al. found that the TCM Bu-Shen-Jian-Pi-Fang could inhibit tumor proliferation by enhancing GLUT-1 related glycolysis and may alter the immune-rejection microenvironment in renal cell carcinoma patients (37). Ginsenoside Re functions as an antioxidant, protecting cardiomyocytes from oxidant injury induced by exogenous and endogenous oxidants, and protects against apoptotic cell death (38, 39). Notably, previous attempts to explore the cancer prevention and therapeutic potential of Lut have systematically indicated its potential as an anticancer agent for various cancers (40). Lut can attenuate antitumor activity and drug resistance via reducing Bcl-2 expression in cancer cells (41). Interestingly, a previous study demonstrated the protective features of Lut against Dox-induced cardiotoxicity, possibly related to its ability of improving Drp1-regulated mitochondrial morphology alteration (42). However, it emphasized on TFEB-mediated mitochondrial regulation and the association between Drp-1 and mTOR, thus ignoring the positive effect of Lut in inhibiting Dox-induced cardiotoxicity in cardiomyocytes and tumor cells.

This study showed that Lut, the core component of *Platycodon grandiflorum*, markedly reduced the level of apoptosis and inhibited the activation of the Bax/Bcl-2/Caspase-3 signaling pathway of cardiomyocytes induced by Dox. Moreover, cytoskeleton damage ruptures cardiomyocytes in Dox-induced cardiotoxicity (43). In this work, Lut protected the cardiomyocyte cytoskeleton damage caused by Dox and maintained the integrity of the cardiomyocyte cytoskeleton. Therefore, cardioprotection from the perspective of protecting the cytoskeleton may be an effective target of Lut for the treatment of Dox-induced cardiotoxicity.

Cardiac autophagic processes lead to ROS overproduction and Δψm dissociation, contributing to mitochondria-mediated apoptosis and death (44, 45). Our present work confirmed that Lut effectively reduced the level of cardiomyocyte oxidative stress and mitochondrial autophagy and inhibited mitochondrial division and the recruitment of Drp-1 phosphorylation. Subsequently, we performed transcriptome analysis to further explore the protective role of Lut in Dox-induced cardiotoxicity. Consistent with previous research (46), our findings indicated the role of Lut in the regulation of mitochondrial morphology, such as Ras protein signal transduction, microtubule cytoskeleton organization, cytoskeletal protein binding, and microtubule binding of molecular function, in GO enrichment analysis. Moreover, we found that the DEGs not only markedly participated in the Hippo/Wnt, AMPK/MAPK, and TGF-β signaling pathways and animal mitophagy process, but were also involved in apoptosis, transcriptional misregulation, and pathways in cancers, such as hepatocellular, breast, gastric, and thyroid cancer. In light of the findings, we carried out follow-up studies on breast cancer cells (4T1 and MDA-MB-231). Notably, Lut exerted a protective effect on Dox-induced cardiotoxicity, improved cardiac function parameters, and enhanced the anticancer therapeutic effects of Dox *in vivo*. Interestingly, combined treatment of Lut and Dox alleviated cardiomyocyte apoptosis but enhanced the apoptosis of breast cancer cells, which were in accordance with previous pharmacokinetics studies highlighting that *Platycodon grandiflorum* combined with Dox can increase the concentration of Dox in the lung and tumor and decrease the concentration of Dox in the heart of breast cancer mice (21). Doubtlessly, the comprehensive findings of Lut and Dox combination in cardiomyocytes and breast cancer cells facilitate its clinical application.

The innovation of this research lies in the mutual verification of *in vivo* and *in vitro* experiments. For the first time, we studied the protective effect of Lut on Dox cardiotoxicity on the basis of a transgenic zebrafish animal model. Second, this study first explored the effect of Lut, the active ingredient of *Platycodon grandiflorum*, on the mitochondrial fusion–division process of cardiomyocytes and the role in the Drp1–Caspase apoptosis signaling pathway. Third, on the basis of transcriptomic sequencing, the mechanism of Lut inhibition of Dox cardiotoxicity was validated in cardiomyocytes and breast cancer cells, which shed light on increasing clinical significance to novel treatment strategies.

Despite the strengths of this study, a number of experimental limitations existed in this study. First and foremost, our study was a cell lines-based study lacking the Dox-induced neonatal rat left ventricle myocyte cardiotoxicity model. Lut retards Dox cardiotoxicity in-depth work is needed in neonatal rat left ventricle myocyte. In addition, the regulation of Lut on Drp-1 phosphorylation and potential binding site remains to be elucidated. Meanwhile, the molecular mechanism of Drp1-dependent mitochondrial autophagy remains unclear. Moreover, the opposite mechanism of Lut-induced apoptosis has not been fully elucidated in cardiomyocytes and tumor cells, more in-depth work is needed for the precise mechanism.

## Conclusion

The protective effect of Lut against Dox-induced cardiac dysfunction is associated with alleviating Drp1-mediated mitochondrial dysfunction. This study first revealed that Lut could potentiate the anticancer effects of Dox in breast tumor cells via the Bax/Bcl-2/Caspase-3 pathway.

## Data Availability Statement

The datasets presented in this study can be found in online repositories. The names of the repository/repositories and accession number(s) can be found below: NCBI; PRJNA763722, PRJNA763517.

## Ethics Statement

The animal study was reviewed and approved by Shanghai University of Traditional Chinese Medicine.

## Author Contributions

YS, FL, MS, and CS conducted the experiments. YS analyzed the data and wrote the manuscript. WH, CW, YX, SZ, HG, JY, and ZZ designed the study and revised the manuscript. DG, YQ, and XH supplied technical support. SL provided all of the reagent. All the authors edited and commented on the manuscript.

## Funding

This study was supported by grants from: National Natural Science Foundation of China (No. 81603629, 81573973, and 81774308).

## Conflict of Interest

The authors declare that the research was conducted in the absence of any commercial or financial relationships that could be construed as a potential conflict of interest.

## Publisher's Note

All claims expressed in this article are solely those of the authors and do not necessarily represent those of their affiliated organizations, or those of the publisher, the editors and the reviewers. Any product that may be evaluated in this article, or claim that may be made by its manufacturer, is not guaranteed or endorsed by the publisher.

## Supplementary Material

Supplementary Figure 1 **(A,B)** Representative TUNEL staining depicting H9c2 and AC16 cell apoptosis after Dox and Lut treatment (200×). White arrows show positive cells.

Supplementary Figure 2 Echocardiographic assay was used to determine the attenuated cardiac structure of Lut on Dox-induced cardiac dysfunction in mice.
