# Supplementary figures and images for: Luteolin Prevents Cardiac Dysfunction and Improves the Chemotherapeutic Efficacy of Doxorubicin in Breast Cancer
Source: Front Cardiovasc Med. 2021 Oct 13;8:750186. doi: 10.3389/fcvm.2021.750186 (PMC8548634; doi:10.3389/fcvm.2021.750186)

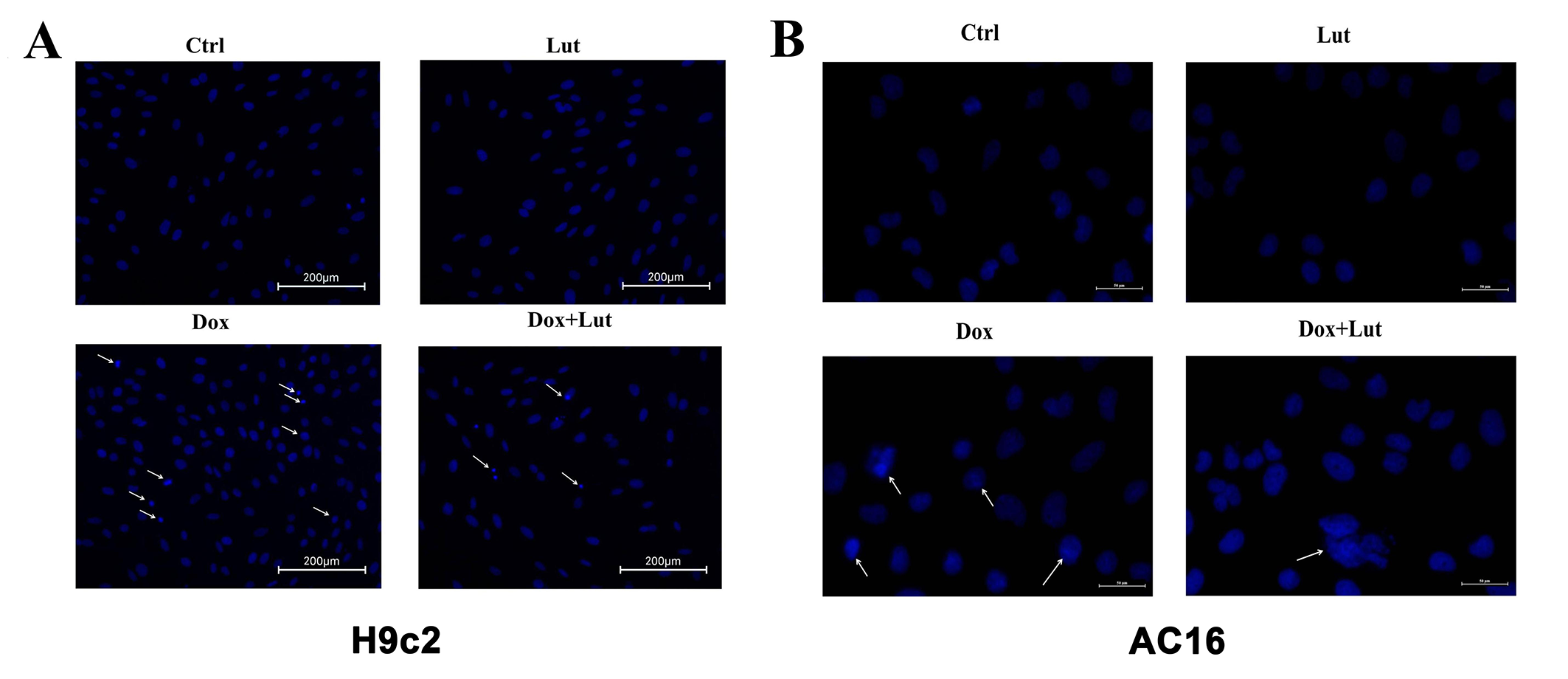

Supplement: Supplementary Figure 1 — (A,B) Representative TUNEL staining depicting H9c2 and AC16 cell apoptosis after Dox and Lut treatment (200×). White arrows show positive cells. [file Image_1.TIF]

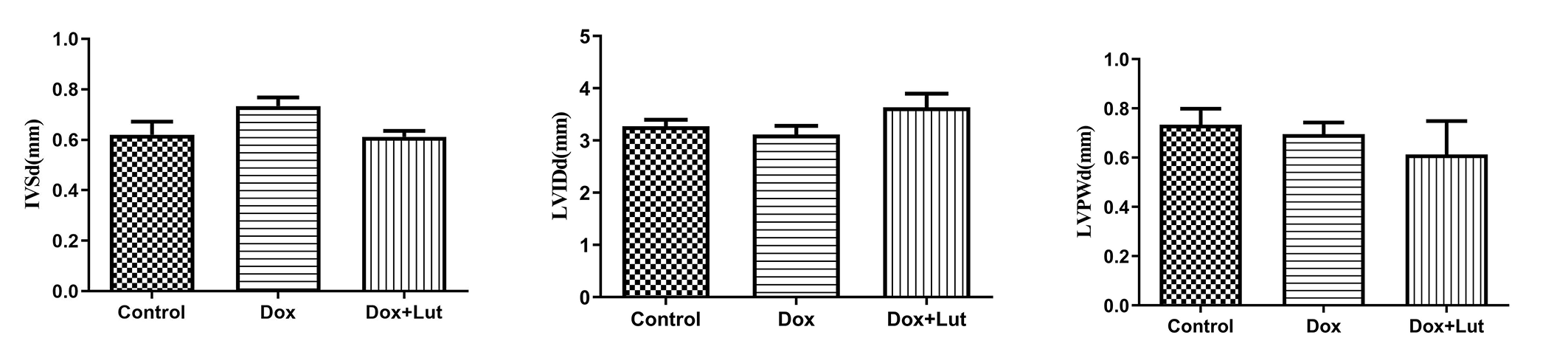

Supplement: Supplementary Figure 2 — Echocardiographic assay was used to determine the attenuated cardiac structure of Lut on Dox-induced cardiac dysfunction in mice. [file Image_2.TIF]
